# Children and young people's experience of parental dementia: A systematic review

**DOI:** 10.1002/gps.5542

**Published:** 2021-04-07

**Authors:** Ilaria Chirico, Giovanni Ottoboni, Marco Valente, Rabih Chattat

**Affiliations:** ^1^ Department of Psychology University of Bologna Bologna Italy

**Keywords:** adaptation, adolescent, adult children, caregivers, child, child development, dementia, family relations, parents, psychological, young adult

## Abstract

**Objectives:**

Most studies have been concerned with the experiences and needs of spouses/partners and adult children of people with dementia. In this review, children and young people's lived experience of parental dementia was investigated. Findings will inform both researchers and professionals in the area of dementia care.

**Design:**

A systematic literature search was performed in CINAHL, PsychINFO, PubMed, Scopus, and Web of Science. A rigorous screening process was followed, and a checklist for qualitative and observational studies was used to evaluate the methodological quality of the studies. Narrative synthesis of the selected articles was carried out.

**Results:**

Twenty‐one studies were included and a synthesis of the literature revealed six themes. The first theme concerned the difficulties in dealing with the diagnosis which was often preceded by a long period characterized by uncertainty, confusion, family distress, and conflicts. The second theme discussed changes in family relationships in terms of the role of children and young people in supporting both parents and keeping family together. The third theme described the impact of caring on children and young people who struggled to balance caring tasks and developmental needs. The fourth theme showed consequences on children and young people's personal lives in terms of education/career and life planning. The fifth theme illustrated main adaptation models and coping strategies. The last theme discussed the need for appropriate support and services based on a “whole family” approach.

**Conclusions:**

The included studies provide the basis for knowledge and awareness about the experience of children and young people with a parent with dementia and the specific needs of support for this population.

## Key points

- Children and young people living and/or caring for a parent with dementia are likely to experience relevant changes in family relationships in terms of providing significant support to both parents and keeping family together
- Parental dementia is likely to affect children and young people's choices, time perspectives and life planning in relation to education/career, mobility, and personal lives
- Children and young people can assume similar levels of caring responsibilities to adult carers, although they may be more vulnerable due to their age and developmental needs
- Appropriate support and care services are urgently needed to suit the needs of this population within a “whole family” approach

## 1 INTRODUCTION

Dementia is one of the major public health challenges in our societies. Worldwide, around 50 million people have dementia, and there are nearly 10 million new cases every year.^1^ It has a huge physical, psychological, social and economic impact on people with dementia and their carers.^2^, ^3^, ^4^ Globally, families provide the majority of care consisting of both instrumental and emotional support for people with dementia.^5^ When symptoms get worse, persons with dementia require more care and supervision by the “entire family unit” with serious consequences on relatives' physical and mental health, well‐being, and social relationships.^6^

Although dementia mainly affects older people, 5%–10% of all cases are estimated to start before the age of 65 years.^7^, ^8^ It is commonly known as “young‐onset dementia” (YOD), and it has an “out of sync” nature when compared with the normal life course.^9^, ^10^, ^11^ Indeed, individuals may still be working, raising families, have dependent children and financial obligations like mortgages.^12^ In the last decades the rise in YOD diagnoses along with the increase of childbirth age and of reconstituted families has led to a higher number of children and young people with a parent with dementia. Actually, it is estimated that one‐third of people with YOD have a child under 18 years.^13^

### 1.1 Young carers

In the last decades, apart from dementia, the issue of living and/or caring for a sick parent at young age has received increased attention due to recent changes in society.^14^ Nowadays, there is no universally agreed international definition of the youth age group, and youth is a more fluid category than a fixed age‐group.^15^ Actually, increasing numbers of young people tend to live in family home at their 30s, and are still financially dependent on their parents, they attend university courses or have temporary jobs.^16^, ^17^

In the case of parental disease, young people may be more vulnerable due to their age and developmental needs that require them to face the new life challenges (e.g., getting a job, attending university, starting their own family) without guidance and support by their parents.^18^, ^19^, ^20^ Statistics indicate that between 4% and 10% of young people care for an ill or disabled parent.^21^, ^22^ The term “carer” (also known as caregiver) refers to anyone who carries out, on a regular and unpaid basis, significant caring tasks for a friend or family member who cannot cope alone because of an illness or other condition.^23^ Caring responsibilities include practical tasks (e.g., cooking, housework, shopping); physical and personal care (e.g., helping someone out of bed, get dressed or with intimate care); emotional support and supervision (i.e., safety and health monitoring). These tasks are often carried out by young people without supervision and assistance, and, in many cases, are comparable to those fulfilled by adult carers.^24^ Furthermore, young people living and/or caring for a sick parent tend to have more mental health problems and more difficulties in behavioral, psychosocial, and academic adjustment than their peers without an ill parent.^23^, ^24^ Since caring can be viewed as a natural extension of family relationships, young people often do not receive adequate support from public authorities, social policy, health, and social services.^23^, ^24^ Despite these findings, most recent studies on the positive impact of caring found that a higher sense of self‐mastery, self‐worth, maturity and empathy are mitigated by the level of support that young carers are provided with.^21^, ^25^ Therefore, if adequate support is available, positive aspects may coexist with the adverse outcomes of caring at young age.^26^

### 1.2 The present study

While most research to date has focused on spouses/partners and adult children of people with dementia, less empirical evidence exists on children and young people's experience of parental dementia as told by themselves. Based on studies with adult samples, parental dementia in families with children and young people leads to greater social and psychological impairment, damage, tension, hardship, and family breakdown than it does in families with adult/middle aged children.^27^, ^28^, ^29^, ^30^, ^31^, ^32^, ^33^ The few available reviews^9^, ^10^, ^11^ focused on the experiences of family caregivers of a relative with YOD. Specifically, the review by Van Vliet et al.^11^ did not include studies on children and/or young people, while in two reviews^9^, ^10^ results were discussed without differentiating among adult and young offsprings. Hence, the aim of this review is to fill this gap by systematically ascertaining the literature on children and young people's lived experience of parental dementia and the psychosocial impact of the disease on their development. Findings will inform both research about the advancement of knowledge and social and healthcare professionals working in the area of dementia care.

## 2 METHOD

### 2.1 Search strategy

The review follows PRISMA guidelines^34^ (Appendix 1). No protocol was published or registered before commencing this review.

The literature selection included a search (Table 1) of articles published only in English until 29 January 2020 in five databases: CINAHL, PsychINFO, PubMed, Scopus, and Web of Science. Full search strategy can be found in Appendix 2. In addition, reference lists from reviewed papers were used to identify additional relevant studies.

**TABLE 1:** Search strategy

Search terms
Child* OR adolescent* OR young adult* OR young caregiver*
2. Parent* with dementia OR parent* with Alzheimer disease OR parent* with frontotemporal dementia OR parent* with dementia, vascular OR parent* with Lewy body disease
3. Life change events OR experiences OR emotions OR adaptation, psychological
4. #1 AND #2 AND #3

The screening process consisted of three stages: duplicate removal, titles/abstracts screening, and full‐text scrutiny. The titles and abstracts of all studies were assessed independently by two reviewers (I. Chirico and M. Valente), and any discrepancy was resolved by a third reviewer (G. Ottoboni) through discussion until an agreement was reached. Finally, full‐paper articles of any relevant titles/abstracts were obtained and reviewed independently by two members of the review team (I. Chirico and M. Valente) with reasons for exclusion annotated; again, any discrepancy was resolved by a third reviewer (G. Ottoboni).

### 2.2 Inclusion/exclusion criteria

In this review, as to reflect current situation in the society, a broad definition of the term “young people” was adopted.^14^, ^15^ Specifically, the term “children” refers to individuals aged 6–10 years, while the term “young people” includes adolescents (11–18 years) and young adults (19–35 years).^16^, ^17^

Articles were included if they focused on: (a) children (aged 6–10 years) and/or young people (aged 11–35 years) (i.e., population of interest); (b) children and/or young people living and/or caring for a parent with dementia (i.e., context of interest); (c) children and/or young people's experience of parental dementia as told by themselves (i.e., outcome of interest); and (d) they reported results of peer‐reviewed research based either on quantitative, qualitative, or mixed method studies. Studies where only a subsample met the eligibility criteria were included if outcomes focused on the population of interest were separately considered and analyzed.

Articles were excluded if they focused on: (a) only spouses/partners and/or adult children (i.e., not population of interest); (1b) children and/or young people living and/or caring for a relative other than their parent (i.e., not context of interest); (2b) children and/or young people living and/or caring for a parent without disease or with other diagnosis than dementia (i.e., not context of interest); (c) causes of dementia, prevalence and incidence, medical considerations, evaluation and assessment of interventions (i.e., not outcome of interest); and (d) they did not report empirical findings. Due to the exploratory nature of this review, there were no restrictions on the type of data to look for and extract.

### 2.3 Quality appraisal

The methodological quality of the included studies was assessed using a quality checklist for observational studies with 23 criteria,^35^ and a quality checklist for qualitative studies with 12 criteria.^36^ If the criterion was met, it was rated with “a+” and unmet with “a−,” and when the criterion was not completely met, it was rated with “+/−.” If the criterion was not applicable, it did not receive any rating. The quality appraisal was carried out independently by two members of the review team (I. Chirico and M. Valente), and interrater reliability was substantial with a Cohen's kappa of 0.75.^37^ After a consensus meeting with a third reviewer (G. Ottoboni) both raters reached full agreement on the quality ratings.

### 2.4 Data extraction and synthesis

A standardized data extraction form^38^ was used to examine the main characteristics (i.e., author/s, year, country, design, population, measures) and key results of the included studies. By using this form, key issues of each study were identified.^39^ An inductive thematic analysis^40^ was used to synthesize data and generate main themes from the included studies. Specifically, themes were compared and grouped to find the most relevant higher level of themes according to similarity across the themes. A label was assigned to each cluster covering similar themes. Each theme captured something important in relation to the overall research question, that is, a key element, domain and dimension of the study phenomenon. The analytical process was followed independently by two researchers experienced in qualitative analysis (I. Chirico and M. Valente), and inconsistencies were resolved by a third reviewer (G. Ottoboni) through discussion until an agreement was reached.

## 3 RESULTS

### 3.1 Study characteristics

**FIGURE 1:**
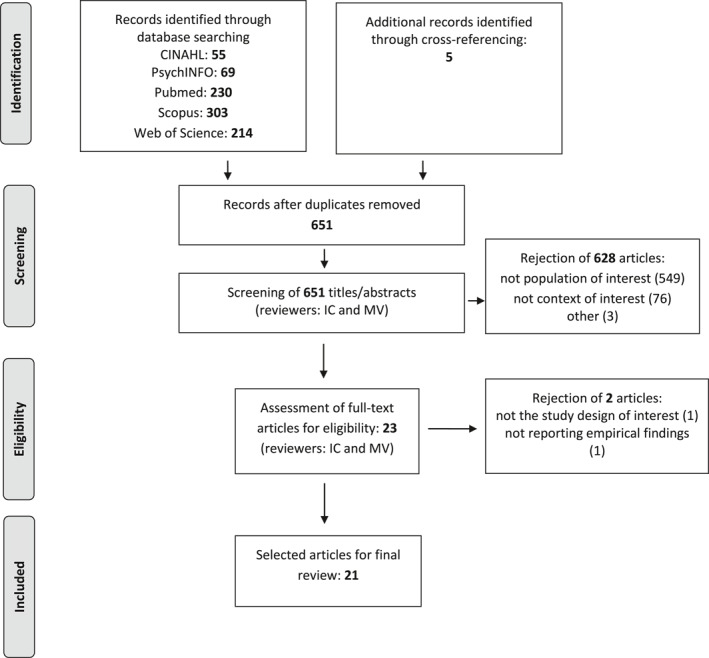
Flow chart showing number of articles selected for systematic review

After duplicate removal, the search identified a total of 651 hits (Figure 1). Of these, 628 records were rejected based on the title or abstract, and two papers^27^, ^41^ were not eligible based on the full‐text scrutiny. Hence, the final sample included 21 studies.

The relevant features of each study (i.e., author/s, year, country, design, population, measures) and key outcomes are presented in Table 2. All studies took place in the West: United Kingdom (*n* = 10), United States (*n* = 4), Norway (n = 3), Australia (*n* = 2), Netherlands (*n* = 1), and Sweden (*n* = 1). As regards the study design, 18 papers used a qualitative approach (10 cross‐sectional and 8 longitudinal), 1 paper used a cross‐sectional quantitative approach, and 2 papers used both quantitative and qualitative measures in a cross‐sectional design.

**TABLE 2:** Main characteristics of the included studies

Author/year	Country	Design	Children			Parents				Measures	Key results
			Gender	Mean age (range)	Living arrangement	Gender	Mean age (range)	Care setting	Diagnosis		
Allen et al. (2009)	UK	Q, CS	F 7, M 5	19 (13–24)	FH 12	M 7	56 (51–64)	H 5, RC 2	AD 3, FTD 2, mixed 1, VD 1	Semi‐structured interview	Five themes: damage of dementia; reconfiguration of relationships; strain; caring; coping. Overarching theme: one day at a time
Aslett et al. (2017)	UK	Q, CS	F 3, M 2	31 (23–35)	OH 3, FH 2	F 2, M 3	60 (52–65)	H 4, RC 1	AD 3, FTD 2	Semi‐structured interview	Five themes: changes in relationships with YOD parent; shifts in role and responsibilities; concern for the nonaffected parent; the need for participants to be supported; the personal impact to self of the parental YOD diagnosis
Barca et al. (2014)	NO	Q, CS	F 12, M 2	NS (20‐35)	OH 14	F 12, M 2	NS (45– 60+)	H 3, RC 11	AD 6, FTD 4, mixed 1, NS 3	Semi‐structured interview	Two themes: experiences in social relationships; experiences and needs related to services.
Davies et al. (2000)	US	MM, CS	F 14, M 6	28 (16–34)	OH 18, FH 2	20 NS	NS	H 20	AD 20	Questionnaires: Symptom Checklist‐90 (SCL‐90), Knowledge Questionnaire (KQ), Self‐Control Schedule (SCS); Semi‐structured interview	SCL‐90: highest scores on interpersonal sensitivity, depression, anxiety and hostility; KQ: 72% average score of correct answers; SCS: skills for managing adverse life events were in the normal range. Four stages of sequential resolving: awareness, explanation, attribution, integration
Denny et al. (2012)	US	QT, CS	F 17, M 7	NS (18–35)	NS	F 9, M 14	NS	NS	FTD 23	Self‐completed online survey	Diagnosis: 52.4% unaware of the name of their parent's diagnosis. Most common sources of information: well parent (23.8%), parent and Internet (23.8%), Internet (19.0%). Emotions: sadness, anger, scariness, and confusion. Most supportive activities included being with friends, sports, music, staying busy, video games, and staying away from home. Caregiving: 57.9% provided regular or significant care. Some felt stressed, annoyed, angry, while others responsible, helpful and grown up. Most difficulties: behavior problems, loss of past relationship, cognitive symptoms and caregiving. Impact: increased closeness of family relationships and growth in self‐confidence. Support: 72.2% interested in meeting children or teens with similar experience
Gelman and Rhames (2016)	US	Q, CS	F 5, M 3	18 (15–20)	FH 8	M 4	NS	H 4	AD 3, FTD 1	Semi‐structured interview	Three themes: abrupt interruption/disruption of child's developmental course; adaptation, coping and growth; lack of YOD information and relevant services
Hall & Sikes (2017)	US	Q, L	22 NS	NS (6–31)	NS	NS	NS	NS	NS	Two to three unstructured interviews over 18 months	Three themes: the process of narrating dementia; “taboo” subjects; portrayals of dementia by people without dementia
Hall and Sikes (2018)	US	Q, L	F 16, M 4	22 (8−31)	OH 11, FH 6, NS 3	F 8, M 10	NS	NS	FTD 8, VD 2, LB 1, PCA 1, NS 6	Two to three unstructured interviews over 16 months	Three themes: something is amiss “you don't normally put three and a half spoons of sugar in your tea”; navigating the pathway; the unpredictability of dementia
Hall and Sikes (2018)	US	Q, L	F 18, M 4	20 (6–31)	OH 11, FH 8, NS 3	F 8, M 11	NS	NS	FTD 8, VD 2, LB 1, PCA 1, NS 7	Up to three unstructured interviews over 12 months. Sessions with children under‐10s involved activities such as “my day,” timelines and family trees	Three themes: disruption to existing family practices; continuities; reconceptualization of relationships
Hall and Sikes (2020)	US	Q, L	F 19, M 4	20 (6‐31)	OH 11, FH 9, NS 3	F 9, M 13	NS	NS	NS	Up to three unstructured interviews over 12 months. Sessions with children under‐10s involved play, drawing, family tree and ‘my day’ storytelling activities	Three themes: constructing the life course; being betwixt and between; managing the betwixt and between.
Hutchinson et al. (2014)	AU	Q, CS	F 11, M 1	24 (19–33)	NS	F 7, M 5	NS	NS	NS	Semi‐structured interview	Four themes: the emotional toll of caring; keeping the family together; grief and loss; psychological distress
Hutchinson et al. (2016)	AU	Q, CS	F 11, M 1	24 (19–33)	NS	F 7, M 5	NS	NS	NS	Semi‐structured interview	Three themes: invisibility; connectivity; being empowered
Johannessen et al. (2015)	NO	Q, CS	F 9, M 5	24 (18–30)	OH 11, FH 3	F 5, M 9	NS	NS	NS	Semi‐structured interview	Four themes and related metaphors: the development and course of the dementia: “my parent is sliding away”; feelings: “emotional chaos”; the transformed relations: “becoming a parent to my parent”; the provision of public services: “a battle”
Johannessen et al. (2016)	NO	Q, L	F 9, M 5	24 (18–30)	T1: OH 11, FH 3; T2: OH 12, FH 1 (dropout 1)	F 5, M 9	61 (57–66)	T1: H 9, RC 5; T2: H 6, RC 7(dropout 1)	NS	Two semi‐structured interviews (T2: 1 year later)	Two themes: detachment; resilience
Lövenmarck (2019)	SE	Q, CS	3 NS	NS (15–25)	NS	3 NS	NS	NS	NS	Discourse analysis of blogs written by participants who had grown up/lived with a parent diagnosed with dementia (TOT = 371 posts over 6–9 years)	Three themes: parent to your parent(s); orphan with parents; time traveler stuck in time
Millenaar et al. (2014)	NE	Q,CS	F 8, M 6	21 (15–27)	FH 14	F 3, M 8	53 (47–62)	H 11	AD 5, FTD 4, VD 1, NS 1	Semi‐structured interview	Three themes: the impact of dementia on daily life; coping with the disease; the need for care and support
Nichols et al. (2013)	US, CA	Q, CS	F 10, M 4	14 (11–18)	FH 14	F 1, M 6	NS	H 7	FTD 7	Focus group	Seven themes: emotional impact of living with a parent with FTD; caregiving; coping; symptoms of FTD; diagnosis; relationships; support
Sikes and Hall (2016)	UK	Q, L	F 16, M 3	22 (8–31)	OH 10, FH 6, NS 3	F 8, M 9	NS	NS	FTD 7, VD 2, LB 1, PCA 1, NS 6	Unstructured interviews (at least two)	One theme: not the same person narratives
Sikes and Hall (2017)	UK	Q, L	F 18, M 4	20 (6–31)	OH 11, FH 8, NS 3	F 8, M 11	NS	NS	AD 7, FTD 8, VD 2, LB 1, PCA 1	Unstructured interviews (at least two) over 18 months. Sessions with children under‐10s involved play, drawing, family tree and “my day” storytelling activities	Six themes: diagnosis; ongoing loss; life on hold; missing landmark events; envy; coping or not
Sikes and Hall (2018)	UK	Q, L	24 NS	NS (6–31)	NS	NS	NS	NS	NS	Up to three unstructured interviews over 18 months. Sessions with children under‐10s involved play, drawing, family tree and “my day” storytelling activities	Five themes: locating parental dementia with reference to educational milestones; dementia‐specific challenges; education as an escape/coping mechanism; dementia and educational choices; educational institutions' responses
Svanberg et al. (2010)	UK	MM, CS	F 6, M 6	14 (11‐18)	NS	F 2, M 7	NS	H 5, RC 2 (deceased 2)	AD 5, FTD 3, VD 1	Questionnaires: Recent Mood and Feelings Questionnaire (MFQ), Zarit Burden Interview‐short (ZBI‐short), Resilience Scale (RS); Semistructured interview	MFQ: 33% had a mood disorder; ZBI‐short: 58% high levels of burden; RS: 83% moderate levels of resilience. Four themes: discovering dementia; developing a new relationship; learning to live with it; going through it together. Three stages of adaptation: grief, emotional detachment, increased maturity

Abbreviations: AD, Alzheimer’s disease; AU, Australia; CA, Canada; CS, cross‐sectional; F, females; FH, family home; FTD, frontotemporal dementia; H, home; L, longitudinal; LB, Lewy bodies; M, males; MM, mixed methodology; NE, Netherlands; NO, Norway; NS, not specified; OH, own house; PCA, posterior cortical atrophy; Q, qualitative; QT, quantitative; RC, residential care; SE, Sweden; UK, United Kingdom; US, United States; VD, vascular dementia.

Child sample sizes varied from 3 to 24 participants per study with a higher number of females; mean ages varied from 14 to 31 years. For what concerns living arrangements, no information was provided in seven studies; the majority of studies sampled young people mostly living in their own house (*n* = 10), while in a few studies (*n* = 4) all participants lived in the family home.

As shown in Table 2, fewer details were provided on parental samples. Sample sizes varied from 3 to 23 participants per study with the majority of males. Based on the data available in four studies alone, mean ages varied from 45 to 66 years. No information about the diagnosis was provided in eight studies. As regards the other ones, most of them sampled people with several diagnoses that mostly were Alzheimer's disease and frontotemporal dementia (*n* = 10) while only few studies had homogeneous diagnostic groups: Frontotemporal dementia (*n* = 2) and Alzheimer's disease (*n* = 1). Based on the data available in nine studies alone, most parents lived in their own house. Finally, for what concerns study measures, interviews were used in qualitative studies: semi‐structured (*n* = 10), unstructured (*n* = 7), and focus group (*n* = 1); the only quantitative study used an online survey while the remaining two studies used a mixed‐methodology consisting of questionnaires and semi‐structured interviews.

### 3.2 Methodological aspects

Most qualitative studies scored high and the main weaknesses were concerned with the limited description of the sample and/or sampling method (Table 3). Specifically, the inclusion and exclusion criteria were not always specified, and not every study explicitly described participant characteristics (e.g., age, living arrangement, parental diagnosis). Description of how sampling was undertaken and justification for sampling strategy were not always reported making it difficult to evaluate the quality of the sample. Moreover, relevant aspects related to the analytic approach were sometimes omitted^42^, ^43^, ^44^ or not fully described. For example, how coding systems evolved, if data were managed by software package or by hand, evidence of more than one researcher involved in the data analysis were not always reported. Findings were discussed in a narrative fashion with an extensive use of field notes entries/verbatim interview quoted. Moreover, they were framed within the social/physical and interpersonal contexts of data collection. Conversely, information about researcher reflexivity was omitted^42^, ^45^, ^46^, ^47^ or not fully provided. Specifically, researchers did not always make explicit their potential influence on the research process and if/how related problems were dealt with.

**TABLE 3:** Quality assessment of qualitative studies

	Author (year)																	
	Allen et al. (2009)	Aslett et al. (2017)	Barca et al. (2014)	Gelman and Rhames (2016)	Hall and Sikes (2017)	Hall and Sikes (2018)	Hall and Sikes (2018)	Hall and Sikes (2020)	Hutchinson et al. (2014)	Hutchinson et al. (2016)	Johannessen et al. (2015)	Johannessen et al. (2016)	Lövenmarck (2019)	Millenaar et al. (2014)	Nichols et al. (2013)	Sikes and Hall (2016)	Sikes and Hall (2017)	Sikes and Hall (2018)
Clear statement of, and rationale for, research question/aims/purposes	+	+	+	+	+	+	+	+	+	+	+	+	+	+	+	+	+	+
Study thoroughly contextualized by existing literature	+	+	+	+	+	+	+	+	+	+	+	+	+	+	+	+	+	+
Method/design apparent and consistent with research intent	+	+	+	+	+	+	+	+	+	+	+	+	+	+	+	+	+	+
Data collection strategy apparent and appropriate	+	+	+	+	+	+	+	+	+	+	+	+	+	+	+	+	+	+
Sample and sampling method appropriate	+/−	+/−	+	+	+/−	+	+	+/−	+/−	+/−	+/−	+	+/−	+	+	+	+	+/−
Analytic approach appropriate	+	+	+	+	+/−	−	−	+/−	+	+	+	+	+	+	+	−	+/−	+/−
Context described and taken account of in interpretation	+	+	+	+	+	+	+	+	+	+	+	+	+	+	+	+	+	+
Clear audit trail given	+	+	+	+	+	+/−	+/−	+	+	+	+	+	+	+	+	+/−	+	+
Data used to support interpretation	+	+	+	+	+	+	+	+	+	+	+	+	+	+	+	+	+	+
Researcher reflexivity demonstrated	+	+/−	−	+/−	+	+/−	−	−	+/−	+/−	+/−	+	+	+	+	+	+	−
Demonstration of sensitivity to ethical concerns	+	+	+	+	+	+/−	+/−	+	+	+	+/−	+	+	+	+	+	+	+
Relevance and transferability evident	+	+	+	+	+	+	+/−	+	+	+	+	+	+	+	+	+	+	+

Abbreviations: +, criterion met; +/−, criterion partly met; −, criterion unmet.

For what concerns the three observational studies, several methodological concerns need to be raised (Table 4). Participants were not representative of the population, sample sizes were small and inclusion and exclusion criteria were not always fully described.^48^, ^50^ Moreover, there was a lack of control for possible confounders. The articles often included children of only parents with specific diagnoses such as Alzheimer's disease^50^ or frontotemporal dementia^48^ and it was unclear how the diagnosis was established. Research hypotheses were always omitted thus making it difficult to interpret the results. Finally, the type of study was never mentioned and outcomes were sometimes neither validated^49^ nor clearly described as means and standard deviations were not reported,^50^ and response rates were not available in two studies.^49^, ^50^

**TABLE 4:** Quality assessment of observational studies

	Author (year)		
	Davies et al. (2000)	Denny et al. (2012)	Svanberg et al. (2010)
Accurate and appropriate outcome measures in all participants	+	+	+/−
Adjustment for confounding	−	−	−
Case/control recruited from the same population (or appropriate alternative)	NA	NA	NA
Appropriate statistical tests used	+	+	+
Participants representative of population	−	−	−
Potential confounders described	−	−	−
Recruitment of case/control over the same time frame (or similar point of disease/illness/treatment)	NA	NA	NA
Participants'characteristics described (age, sex, diagnosis, relationship between patient and carer)	+	+	+
Numerical description of important outcomes given	+/−	+	+
Outcomes clearly described	+/−	+	+
Response/non‐response rate described	−	+	−
Clear case/control definition	NA	NA	NA
Power calculation used	−	−	−
Losses and completers described	NA	NA	NA
Reliable assessment of disease state	+	−	−
Clear inclusion/exclusion criteria	+/−	+/−	+
Clear hypothesis	−	−	−
Reported probability characteristics	+/−	−	+
Type of study stated	−	−	−
Main findings described	+/−	+	+
Disclosure of funding source	+	+	−
Conclusions supported by findings	+/−	+	+
Statistical tests of heterogeneity	NA	NA	NA

Abbreviations: +, criterion met; +/−, criterion partly met; −, criterion unmet; NA, not applicable.

### 3.3 Findings

#### 3.3.1 Diagnosis

Participants reported that diagnosis was often a lengthy process causing confusion and uncertainty and exacerbating family distress and conflicts.^42^, ^51^, ^52^, ^53^, ^54^, ^55^ Lack of diagnosis or misdiagnosis had financial implications for the family as well.^51^, ^52^, ^54^, ^55^ Although receiving a diagnostic label was usually perceived as a “shock” and it was very difficult to accept, it represented an important marking point to understand the disturbing changes in the personality and behaviors of the parent with dementia.^53^, ^54^, ^55^, ^56^ Diagnosis was also useful to receive support from health and social services, and an exemption from social roles and expectations.^51^, ^53^, ^54^, ^55^ The survey based study of Denny et al.^48^ with 24 young adults found that roughly half of the sample (47.6%) had been told the name of their parent's diagnosis, 52.4% had not. Emotions were sadness, anger, fear, and confusion. When asked “where or to whom did you turn with questions about the diagnosis?” the most frequent answers included the well parent (23.8%), the parent and Internet (23.8%), and the Internet alone (19.0%). 23.8% of the sample responded they had no one to turn or preferred not to talk.

#### 3.3.2 Family relationships

Significant role changes occurred in terms of parentification/role reversal, that is, participants took on parent‐like responsibilities for their parents with dementia while adapting their own lives to parental needs.^43^, ^49^, ^50^, ^51^, ^56^, ^57^ Participants were “lost in the chaos”^51^ as their parents gradually lost interest in them, exhibited aggressive and embarrassing behaviors and they did not know how to react. Participants experienced confusion, disorientation, fear, and sadness which rose, in some cases, to the level of emotional trauma.^51^, ^56^, ^58^ They disliked the parent who had become, and some spoke of “relief” for them and “release” for their parent when death finally came.^53^, ^55^

The relationship among participants and their parents with dementia was usually characterized by “latent grief,” and an ongoing and unmitigated loss as the condition worsened.^45^, ^48^, ^49^, ^51^, ^52^, ^58^, ^59^ Parents were physically present but emotionally absent, and they were no longer available in any meaningful parental way.^45^, ^46^, ^53^, ^54^, ^55^, ^58^ Participants told about their unique experience of grieving the loss of their parents due to a disease that, although fatal like other illnesses, society stigmatized creating an aura of uncaring.^59^ Several concerns for the well parent were raised and they often assumed the role of “protectors” in the attempt to reduce the burden on their healthy parents.^45^, ^51^, ^52^, ^54^, ^57^ In the study of Lövenmarck^60^ they became orphans with parents, that is, parents to both of their parents in order to support and keep them healthy for as long as possible.

In the study of Hall and Sikes^43^ focus was on family practices. Dementia significantly affected the ways of doing family in a process of constant change and adaptation with uncertainty about timeline and rate of change. Ways of spending time together, such as days out and holidays, were no longer possible and conversations also changed in their content and quality. They mostly regarded parental interests and issues or, in worst cases, parents stopped to communicate or to recognize their children. Participants also contemplated future lost elements of family display such as a mum absent from wedding dress shopping with a daughter, or a dad missing at graduation. However, there were also some practices where relationships were maintained although the onus was placed on the participants as main agents in the relationship. This happened when small interactions took place, or when parents remembered their child's name or said “I love you.” Tensions and disruptions in the quantity and quality of the extended family connections occurred with reactions of denial, distancing and lack of involvement.^58^

#### 3.3.3 Caring

Participants had extensive caring responsibilities including physical support of the parent such as helping with feeding and ambulating, household chores but also companionship, talking with healthcare professionals, driving for errands.^48^, ^54^ They provided not only instrumental care, but also emotional care to their healthy parents as to comfort them, mediate conflicts and keep the family together.^57^, ^59^

Caregiving took an emotional toll on the participants as they struggled to find a balance between being a young person and a caregiver.^48^, ^51^, ^52^, ^54^, ^59^ The impact of caring varied from ongoing stress to a medically diagnosed mental health condition that then compounded their disability such as depression, anxiety, psychosis, obsessive‐compulsive disorder, self‐harm, alcohol and substance addiction, suicide ideation.^49^, ^50^, ^51^, ^59^ Some positive aspects associated with the experience of caring were also reported such as increased maturity and the opportunity to spend time with their parent.^48^, ^54^

Levels of participants' burden were different depending on the previous family relationships, the family reorganization after diagnosis as well as the nature and severity of parental symptoms and the speed of decline.^45^, ^52^, ^58^ They were higher in families where participants lived with a single parent with dementia or when families were denied social security, the healthy parent struggled to find a job or was dealing with his/her own emotional issues.^59^ On the contrary, levels of burden were lower in families where the well parent was the primary caregiver, and managed the situation in ways participants found reasonable, or when family members like siblings collaborated and shared the tasks.^45^, ^48^ Furthermore, those who lived outside of the home seemed to be less influenced by their parent's illness than their peers living at home.^45^

#### 3.3.4 Personal life

Parental dementia strongly affected participants' choices, time perspectives and life planning in relation to education/career, mobility and personal lives.^43^, ^46^ Participants felt their lives were in “limbo,” in “betwixt and between”^46^, p. 245. For some, the future was a source of deep anxiety, while others were unable to contemplate life beyond their parents' illness, and life was put on hold.^52^ They had fewer possibilities to focus on their development and were not able to develop an identity as they would have liked during their youth.^52^, ^56^, ^58^, ^60^

Participants' concerns and lack of concentration sometimes hindered progress in their studies and career; some had interrupted their studies or work to return home and take care of their parents.^45^ Educational choices were done, at least partly, because of parental dementia or decisions not to move were based on the need to stay close to their family and spend time with the parent with dementia.^47^ Many participants at school pointed out the disparity in being acknowledged as young carers due to the lack of awareness about YOD among teachers.^47^, ^61^ When the well parent informed the teaching staff about the situation at home, schools often did not accommodate the timetable to students' needs by distance learning for example. In most cases teachers did not understand participants' experience, the schools did not sensitively match their response to students' needs through the provision of professional help like a counselor. Furthermore, when the condition was known, students felt marked as different, stigmatized and bullied by their schoolmates. Conversely, participants received support at college and university when they informed tutors or sought help from counseling services. However, in this context, very few students revealed their situation largely because of ignorance of dementia exacerbated by media focus on dementia as Alzheimer's and a disease of older people.^47^

#### 3.3.5 Adaptation and coping

Some models of young people's adaptation to parental dementia were proposed. For example, Davies et al.^50^ described four stages of sequential resolving in Alzheimer's disease: awareness, explanation, attribution, and integration. At first, functional and psychological changes in the parents were not global in nature and were easily denied or attributed to another cause like stress. Over time symptoms were no longer dismissible and, at this stage, usually family members worked together to bring each other's awareness of their relative's symptoms. At the explanation stage participants were actively involved in looking for medical diagnostic work and assistance. If the diagnosis was not accepted, they looked for a second opinion and, in extreme cases, they became lay experts as to self‐protect or accept the disease and prepare for the future. At the attribution stage it was crucial to redefine the parent as a patient, grieve the person who was, and adapt to the changes in family while also carrying on with his/her own life. The fourth stage was when young people had to integrate the parent and the patient into the same person while retaining both the memories of the parent who was and the present experiences of the parent. Allen et al.^51^ saw evidence in participants' accounts of awareness, explanation and attribution but, instead of integration, they found the development of grief. It might be due to the study sample, that is, only 10% of Davies et al.'s^50^ participants lived at home with their parents and, probably, the rest of them found it easier to distance themselves emotionally.

Svanberg et al.^49^ described three phases of young people's adjustment: grief for the parent before dementia, emotional detachment from the parent, and becoming a grown up. She found that, only when the disease was accepted, a new relationship developed with the parent with dementia viewed as a different person like a child. This allowed emotional detachment since difficulties were blamed on dementia rather than on the parent leading the participant to “learn to live with it,” and “go through it together” (p. 745) as an adult equal to the other parent. This autonomy and independence resulted in feelings of more grown up even though it could have been premature. Indeed, some participants did not see themselves as carers, and did not feel they had sufficient responsibilities to warrant this label.^45^, ^51^

As regards coping strategies, in the study of Allen et al.^51^ young people reported mainly emotion‐focused coping, but also problem‐focused coping in that they helped with care, supporting siblings, and obtaining formal help. Maladaptive strategies were denial, social and emotional withdrawal, smoking, abuse of alcohol and self‐harm. In Aslett et al.'s^52^ young people employed problem‐focused coping strategies when duties were tangible, while a lack of mastery or control was associated with feelings of hopelessness. Fears over genetic risk were managed by adopting mindful coping strategies that focused on living in the moment.^51^, ^52^ Some participants grew up stronger, excelled in school, involved into academic and extracurricular activities as ways of coping and distraction; others told that this experience reinforced their faith which, in turn, helped them to cope better.^49^, ^50^, ^58^ Other supportive activities included being with friends, sports, music, staying busy, video games, and staying away from home.^48^

Millenaar et al.^57^ found that, especially at the beginning, young people adopted avoidant ways of coping because they did not know how to deal with the situation. Talking about the disease and their difficulties with the family was difficult, and some participants did not reveal their feelings as to protect themselves from the possibility of emotional hurt and, instead, focused on other aspects of their lives as a distraction. Adaptable participants were those who stayed positive and were patient in adjusting to their parents' needs. It was also important for them to rest and to attempt to live their lives as normally. When problems occurred at home, they opted for an open communication with their family or, alternatively, they were happy to confide in someone in a select group of people other than their well parent to avoid burdening him/her.

#### 3.3.6 Care and support

Participants felt neglected by family, friends, health, and social professionals. They experienced discrimination and marginalization due to the way services were designed and delivered.^44^, ^45^, ^46^, ^47^, ^48^, ^49^, ^52^, ^54^, ^56^, ^59^, ^60^, ^61^ On the one hand, they felt isolated from the community but, on the other hand, they often did not reveal their situation as not to draw attention of their situation to social services and to avoid the stigma.^59^, ^61^

Participants were mainly concerned about the needs of their parents, and they were more likely to ask for help after watching their healthy parents do the same.^54^ Assistance with activities of daily living and mobility was needed as well as a better support for their family. The latter one should allow the well parent to retain his/her role, and children and young people to assume more age appropriate roles and be successfully engaged into school and work.^45^, ^49^, ^54^

Since the lack of personal experience acted as a barrier to communication, participants preferred to talk with at least one professional who was familiar with their situation and with knowledge of the disease and available services. This was preferred to support provided by sporadically visiting healthcare professionals who were felt too impersonal.^54^, ^60^ Similarly, being involved with peer support groups who had personal experience of dementia was considered valuable in managing stress, burden, guilt and increasing their understanding of dementia.^45^, ^52^

Participants often used the Internet and social media to obtain information and advice, and to make contact with others with similar experiences.^44^, ^60^ Hence, they appreciated the use of technology (e.g., online forums, blogs) to exchange personal experiences, get information and practical guidance while reducing their feelings of isolation.^52^, ^60^ In a similar way, they required support at school if they had to fulfill their educational potential, enjoy the social opportunities and have personal goals.^45^, ^47^

## 4 DISCUSSION

If literature is largely concerned with the needs of spouses and adult children of people with dementia, in this review a systematic overview of the literature on children and young people's lived experience of parental dementia was provided. The main themes concerned their experience of diagnosis and caring, the impact of dementia on their family and personal life, main adaptation models and coping strategies, and needs for care and support. These results are unique to this population for different reasons. Children and young people, although assuming similar levels of caring responsibilities to adult carers, can be more vulnerable due to their age and developmental needs. Furthermore, these results cannot be assumed to reflect of other caring relationships such as grandparents. Indeed, a parent with dementia, due to the peculiar characteristics of the disease, is gradually no longer available in any meaningful parental way during a delicate phase of their children's development. Another peculiarity concerns the “invisible” nature of their experience and lack of specialist support and care.

At the onset of parental symptoms and, throughout the disease trajectory, participants were “lost in the chaos”^51^ and accepting the diagnosis was difficult. Over time participants had increasing responsibilities as adults, while they experienced a “latent grief”^54^ as their parent was no longer emotionally available although physically present. At the same time, they provided instrumental and emotional care to both parents as to keep the family together. In this scenario, they experienced similar levels of emotional distress to those observed in the adult carers.^49^, ^50^, ^51^, ^59^

Participants had to deal with the unpredictability of dementia and their plans in relation to education/career, mobility, and personal lives were strongly influenced by their parent's condition. Some had worsened in school or had even given up their studies. At school they were stigmatized and their educational needs in relation to parental dementia were neglected.^44^ At the same time support from extended family and friends was greatly disturbed.^56^, ^62^ Hence, their feelings of neglect and marginalization were similar to those experienced by young carers of parents with other mental disorders.^59^, ^61^ It is important to note that caring at young age was sometimes viewed as a natural extension of family relationships.^49^

Participants described the existing services as silo‐based and inappropriate for their needs. A “whole family” approach should be based on a strong cooperation among adult, child, health, and voluntary sectors. This joint work should be facilitated by a case manager with knowledge of the family situation, and capable to organize specific care as to alleviate the burden on the well parent which, in turn, would decrease the pressure on their children.^60^ Professionals should have appropriate interdisciplinary training with knowledge of dementia, consequent changes in family dynamics, and children and young people's developmental needs.^44^, ^45^, ^46^, ^52^, ^56^

It is nevertheless important to point out that not all of them will experience these difficulties depending on the availability of support.^47^, ^58^, ^59^, ^61^ Broader societal views and a greater public understanding may promote increased support at the policy level, and better emotional and practical support for children, young people and their families.^45^, ^58^, ^63^ Psychoeducation, face‐to‐face groups, online forums and/or blogs may suit the needs of support for this population.^52^, ^54^

### 4.1 Limitations

Although the review was rigorous, the gray literature was not included as well as articles published in languages other than English. The study samples were small, were not always fully described and most studies had a cross‐sectional design. They had a fairly wide age range and did not clearly differentiate among children and young people's experience, stages in young people's development and in the dementia pathway, different family contexts and parental diagnoses. The area of social relationships including romantic relationships was not fully addressed as well. Furthermore, all the included studies were conducted in the Western countries making it impossible to generalize findings to different countries and healthcare systems. Future research could focus on obtaining longitudinal data on different phases of child development and parental disease stages, family structures, and healthcare systems.

### 4.2 Conclusions

Findings should contribute to raising awareness about the peculiar and often “invisible” experience of young people with a parent with dementia. Furthermore, they should inform research, practice, program development, and policymakers in the area of dementia care.

Around the world, there are already some good practices (nondisease specific) including young carer groups, forums, some awareness campaigns, ICT and web‐based interventions.^25^ Preventive actions should avoid the negative consequences of caring at young age which, in turn, would save society the costs of increased healthcare.^25^, ^64^, ^65^ Since dementia caring can be variable over time, the system based on a “whole family” approach should be flexible and proactive focused on specific needs at specific times. At school, a clear framework of support should be embedded into the school policy. Chances for students to reveal their situation are necessary along with trainings for teachers on recognizing and supporting their needs. All initiatives should be based on children, young people and their family's involvement in decision‐making as to develop tailored interventions suited to the particular needs of this population.

## CONFLICT OF INTERESTS

The authors declare that there are no conflict of interests.

## AUTHOR CONTRIBUTIONS

Rabih Chattat conceptualized the study. Ilaria Chirico and Marco Valente acquired the data and performed the data analysis. Giovanni Ottoboni supervised the process. All authors interpreted the data. Ilaria Chirico wrote the first draft of the manuscript. Agreement has been reached for all aspects of the manuscript in ensuring that questions related to the accuracy or integrity of any part of the work are appropriately investigated and resolved. All authors critically revised the manuscript and agreed to the published version of the manuscript.

## Supporting information

Supplementary Material 1

Supplementary Material 2
