# Supplementary material for: Children and young people's experience of parental dementia: A systematic review
Source: Int J Geriatr Psychiatry. 2021 Apr 7;36(7):975–92. doi: 10.1002/gps.5542 (PMC8252592; doi:10.1002/gps.5542)
Supplement: Supplementary file 2 — Supplementary Material 2 [file GPS-36-975-s002.doc]

**Appendix 2** Full electronic search strategy

| **DATABASE** | **Pubmed** |
| --- | --- |
| **DATE** | **01/29/2020** |
| **STRATEGY** | **#1 AND #2 AND #3** |
| #1 | ("child*"[All Fields] OR "adolescent*"[All Fields] OR (("young"[All Fields] OR "youngs"[All Fields]) AND "adult*"[All Fields]) OR (("young"[All Fields] OR "youngs"[All Fields]) AND "caregiver*"[All Fields])) |
| #2 | (("parent*"[All Fields] AND ("dementia"[MeSH Terms] OR "dementia"[All Fields] OR "dementias"[All Fields] OR "dementia s"[All Fields])) OR ("parent*"[All Fields] AND ("alzheimer disease"[MeSH Terms] OR ("alzheimer"[All Fields] AND "disease"[All Fields]) OR "alzheimer disease"[All Fields])) OR ("parent*"[All Fields] AND ("frontotemporal dementia"[MeSH Terms] OR ("frontotemporal"[All Fields] AND "dementia"[All Fields]) OR "frontotemporal dementia"[All Fields])) OR ("parent*"[All Fields] AND ("dementia, vascular"[MeSH Terms] OR ("dementia"[All Fields] AND "vascular"[All Fields]) OR "vascular dementia"[All Fields] OR ("dementia"[All Fields] AND "vascular"[All Fields]) OR "dementia vascular"[All Fields])) OR ("parent*"[All Fields] AND ("lewy body disease"[MeSH Terms] OR ("lewy"[All Fields] AND "body"[All Fields] AND "disease"[All Fields]) OR "lewy body disease"[All Fields]))) |
| #3 | ("life change events"[MeSH Terms] OR ("life"[All Fields] AND "change"[All Fields] AND "events"[All Fields]) OR "life change events"[All Fields] OR ("experience"[All Fields] OR "experience s"[All Fields] OR "experiences"[All Fields]) OR ("emoting"[All Fields] OR "emotion s"[All Fields] OR "emotions"[MeSH Terms] OR "emotions"[All Fields] OR "emotion"[All Fields] OR "emotional"[All Fields] OR "emotive"[All Fields]) OR ("adaptation, psychological"[MeSH Terms] OR ("adaptation"[All Fields] AND "psychological"[All Fields]) OR "psychological adaptation"[All Fields] OR ("adaptation"[All Fields] AND "psychological"[All Fields]) OR "adaptation psychological"[All Fields])) |
| Filters | English |
